# Returning home to die or leaving home to seek health care? Location of death of urban and rural residents in Burkina Faso and Senegal

**DOI:** 10.1080/16549716.2018.1475040

**Published:** 2018-06-05

**Authors:** Bruno Lankoandé, Géraldine Duthé, Abdramane Soura, Gilles Pison

**Affiliations:** a Center for Demographic Research, Université catholique de Louvain, Louvain-la-Neuve, Belgium; b French Institute for Demographic Studies, Paris, France; c Institut supérieur des sciences de la population, Université de Ouagadougou, Ouagadougou, Burkina Faso; d Museum national d’histoire naturelle, Paris, France

**Keywords:** Location of death, short-term mobility, end of life, place of death, Health and Demographic Surveillance System, Burkina Faso, Senegal

## Abstract

**Background**: In sub-Saharan Africa, the literature on end of life is limited and focuses on place of death as an indicator of access and utilization of health-care resources. Little is known about population mobility at the end of life.

**Objective**: To document the magnitude, motivations and associated factors of short-term mobility before death among adults over 15 years of age in Burkina Faso and Senegal.

**Methods**: The study was based on deaths of adult residents reported in three Health and Demographic Surveillance System (HDSS) sites in urban (Ouagadougou) and semi-rural areas (Kaya) of Burkina Faso, and rural areas of Senegal (Mlomp). After excluding deaths from external causes, the analysis covered, respectively, 536 and 695 deaths recorded during the period 2012–2015 in Ouagadougou and Kaya. The period was extended to 2000–2015 in Mlomp, with a sample of 708 deaths. Binary logistic regressions were used to examine the effects of socio-demographic characteristics on place of death (health facility or not) and location of death (within or outside the HDSS).

**Results**: In Mlomp, Kaya and Ouagadougou, respectively 20.6%, 5.3% and 5.9% of adults died outside the HDSS site. In Mlomp and Kaya, these deaths were more likely to occur in a health facility than deaths that occurred within the site. The reverse situation was found in Ouagadougou. Age is the strongest determinant of mobility before death in Mlomp and Kaya. In Mlomp, young adults (15–39) were 10 times more likely to die outside the site than adults in the 60–79 age group. In Ouagadougou, non-natives were three times more likely to die outside the city than natives.

**Conclusions**: At the end of life, some rural residents move to urban areas for medical treatment while some urban dwellers return to their village for supportive care. These movements of dying individuals may affect the estimation of urban/rural mortality differentials.

## Background

According to Gu and colleagues [1], place of death goes through three evolutionary stages in societies. During the first stage, most people die at home because of poor access to health-care resources. In a second stage, deaths in hospitals become more common due to health systems advances. In the last stage, the emphasis shifts to quality of care at the end of life, with the development of home-based care to meet people’s preferences for death at home. During the different stages, cultural norms play a role in determining the place of death.

In developed countries, the public health debate is particularly centred on place of death as an indicator of quality of end-of-life care, with most people preferring to die at home rather than in hospitals or care homes [2]. In some countries such as Japan and Belgium, a large share of deaths still occur in hospitals, while in the USA and Canada, death at home or in nursing homes is tending to replace death in hospital [3–7].

Referring to the three-stage transition model of Gu et al. [1], we assumed that developing countries, in sub-Saharan Africa (SSA) especially, are still in stage 1 or in transition from stage 1 to stage 2. People still face serious health issues, including the rise of non-communicable diseases in a context of endemic infectious diseases and poverty [8]. Prevention and cure of infectious diseases remain a priority. Terminal care is pratically non-existent except in countries which were severely affected by the HIV epidemic such as Zimbabwe, Uganda, South Africa, Kenya [9]. Statistics on place of death (health facility versus home death) are limited because of the lack of exhaustive vital registration systems in most countries [10]. In recent years, however, a few population-based studies have examined where people die, particularly in Eastern and Southern Africa. Among adults aged 15 years and above, the proportion of deaths occurring in hospitals was estimated at 29% in Addis Ababa [11]. In Zambia, a recent study showed that 51% of adults (15–59 years) died in hospital [12]. In Botswana, it was estimated that 64% of adult deaths (18 years and above) occurred in hospitals after excluding deaths from external causes and pregnancy-related deaths [13]. In West Africa, a pioneering study on place of death was conducted by Bado and colleagues [14], in a semi-rural setting of Burkina Faso. The authors showed that for all ages, 45% of deaths occurred in hospital but just 36% among adults aged 15 years and above [14].

It is recognized that socio-demographic, clinical and ecological factors have an impact on place of death [12,15,16]. According to the Behavioral Model of Health Services Utilization developed by Phillips et al. [17], socio-demographic factors such as age, gender, marital status and education have an impact on the ability to obtain care. For instance, in the African context, it may considered inappropriate for elderly people (or their relatives) to seek care for diseases of old age [18]. As found in Addis Ababa and Botswana, the elderly are more likely than young adults to die at home [11,13]. The clinical component in the analysis of place of death refers generally to the cause of death. In the model developed by Phillips et al. [17], the type of disease contributes to defining a patient’s need for medical care. They hypothesize that some diseases such as cancer, renal failure and cardiovascular diseases are difficult to manage at home and will probably lead to hospitalization. Contradictory evidence on the association between cause of death and place of death has emerged from the limited research conducted in SSA. In Addis Ababa, deaths due to cardiovascular diseases occur more frequently in a health facility than deaths due to HIV/AIDS. But in Zambia, no association between place of death and cause of death has been found except for deaths from external causes and from HIV/AIDS. Finally, type of place of residence and living standard are commonly used as a proxy of enabling characteristics for care-seeking. Type of place of residence mostly determines the availability of health-care resources. Home death is more common in rural areas than in urban settings of SSA [12,14]. For example, in Zambia, adults living in urban areas were twice as likely to die in a health facility than rural residents [12]. This underscores rural–urban inequality in access to health-care resources in favour of urban areas.

Despite the various investments made in the provision of health facilities in rural areas of SSA since the Alma Ata conference in 1978, the urban–rural gap in access to health care is still very wide [19,20]. Health facilities and skilled health workers (doctors, nurses, midwives) are concentrated in cities [21]. Furthermore, they offer a variety of services including care for some non-communicable diseases such as diabetes and hypertension that may not be available in rural remote areas. For example, in Senegal, it was estimated that the number of physicians per 1000 population was around 0.07 in rural areas and 2.33 in urban areas [21]. Consequently, some rural residents in need of care must travel to urban areas to seek treatment while urban residents may have local access to health care.

In SSA, however, some urban residents may go back to rural areas at the end of their life to obtain the supportive care they need. In particular, urban dwellers with strong social ties in rural areas, such as rural–urban migrants, may decide to die in their home community. This is likely to happen when there is a little hope of a cure. Some chronic diseases, for instance, cannot be treated effectively, even in the largest city hospitals, as treatments are too costly for both individuals and the state [22]. A recent example is that of rural–urban migrants with HIV/AIDS in urban areas of South Africa who often returned to their village to receive supportive care and to die [23,24]. In addition to the need for supportive care, death far from home may be considered culturally as a ‘bad’ death, as documented in rural Ghana [25]. Communities do not want their members to die alone, far away: in some cases, the bodies of people who die away from home are even repatriated at great expense and buried within the community. To avoid the prospect of being buried away from home, some terminally ill migrants will prefer to go home to die. This was shown in a qualitative study conducted among international migrants from Zimbabwe in South Africa [26].

To sum up, the existing literature suggests that in African countries, the terminally ill are a potentially mobile population. Some rural dwellers may travel to urban areas to seek care and die there, while urban patients in need of supportive care may return to their rural origins and die at home.

The paper aims to document the magnitude, motivations and associated factors of short-term mobility before death among adult residents in rural and urban contexts of West Africa. Regarding motivations, we hypothesized that the mobility of rural residents before death is linked to health-seeking behaviour; these deaths are likely to occur in health facilities. Conversely, the mobility of urban residents may be due to the need for end-of-life supportive care, so that these deaths are likely to occur at home. Finally, to explain mobility before death, we relied on the traditional variables identified in the literature as associated with place of death. But, in addition to these variables, migration status was taken into account in urban areas as we expected rural–urban migrants to be more likely to return to rural areas before death than non-migrants.

The data used come from the Mlomp Health and Demographic Surveillance System (HDSS) in Senegal, and the Kaya and Ouagadougou HDSSs in Burkina Faso. These HDSSs are respectively located in rural, semi-rural and urban areas. Mobility before death was defined on the basis of information on the location of death, i.e. if the death occurred within the HDSS or outside the HDSS. But in the case of the Ouagadougou HDSS, to ignore mobility within the city, the location of death was defined in relation to the entire city. The mobility considered here is quite different from out-migration in its habitual sense, which generally involves a long-term change in place of residence. Here, we were only interested in the final movement of individuals within six months of death.

To the best of our knowledge, this is the first time that mobility before death has been investigated in the context of West Africa. The study provides useful information on the needs of ill and dying people living in urban and rural environments, and on their respective coping strategies. It also has implications for the measurement of spatial health inequalities, and of mortality indicators in particular. In censuses, and Demographic and Health Surveys, which remain the main sources of mortality statistics in West Africa, the death of an adult may be wrongly classified as an urban or rural death.

## Data and Methods

In the context of SSA, data collected in censuses and standard household-based surveys such as the Demographic and Health Surveys (DHS) do not allow a detailed analysis of rural–urban migration in relation to adult health [27,28]. First, these surveys do not collect often information on migration flows between urban and rural areas. Second, the focus is on child and maternal health rather than on adult health. Finally, the few data available on migration concern long-term mobility leading to change in a place of residence. So, capturing short-term mobility and its relation to health is a particular challenge.

The data used here come from three HDSSs of Burkina Faso and Senegal. They will serve as case studies to explore mobility before death in different West African contexts. The first one is the Ouagadougou HDSS in Burkina Faso, which is the only fully urban HDSS of West Africa. Second, the Kaya HDSS will allow an analysis of mobility before death in a semi-rural context of Burkina Faso. The Kaya HDSS is covered by a regional hospital. Finally, the Mlomp HDSS located in rural Senegal offers a contrasting context compared to that of Kaya because the site is covered by a primary health-care facility only. Furthermore, in this HDSS site in particular, information is collected on whether mobility is related to health-care-seeking. Such data allow a better understanding of the reasons for mobility before death in rural areas. While other HDSSs sites exist in West Africa, only data from these three sites were accessible and/or suitable for the analysis conducted here.

The three HDSS sites are members of the International Network for Demographic Evaluation of Populations and Their Health (INDEPTH) and share similarities in terms of methodology [29]. Following an initial census in the area under surveillance, fieldworkers conduct regular household update rounds, and register vital events (births and deaths, migrations and marriages). In case of death, a verbal autopsy (VA) questionnaire is completed with the next of kin to determine the circumstances that led to the death, including the history of the illness and the specific symptoms that preceded death. VA data can be interpreted by physicians or using computer-based methods. In the first method, each VA is reviewed separately by two distinct physicians to determine the probable cause of death. In case of disagreement, the VA is reviewed by a third physician who assigns a consensus diagnosis. Otherwise, the cause of death is categorized as ‘indeterminate’ [30]. The most widely used automated method on INDEPTH sites is the one based on Bayes’ theorem implemented in the InterVA-4 software [31].

The Ouagadougou HDSS was established in late 2008 in five neighbourhoods at the northern periphery of the capital city of Burkina Faso [32]. Two of them (Kilwin and Tanghin) are formal neighbourhoods with full access to public services, while the other three (Nonghin, Polesgo and Nioko 2) are informal settlements (like slums) without access to such services [32]. People living in the Ouagadougou HDSS are mostly from the Mossi ethnic group (90%), which is currently the majority ethnic group in the country. More than half of active adults work in the commerce and construction sectors [33]. The population under surveillance in the Ouagadougou HDSS totalled about 90,000 residents in 2015 and periodic household update rounds are conducted with an average periodicity of 10 months. VA data are interpreted by the InterVA-4 software to determine the probable causes of death. Health-care provision in the city of Ouagadougou is better than in any other location in Burkina Faso, with a private sector representing two-thirds of all care services. In addition, large teaching hospitals offering the country’s highest standards of care are located in the city [34].

The Kaya HDSS was established in late 2007 in the North Central region of Burkina Faso, 100 kilometres from the capital city, Ouagadougou. The site covers the town of Kaya and 18 villages [35]. The follow-up population was estimated at 70,000 inhabitants in 2015. This population lives in semi-urban (70%) and rural (30%) areas. The site is easily accessible from Ouagadougou and is covered by seven health facilities and one regional hospital. Residents are mostly from the Mossi ethnic group and are of Muslim faith. Only half of the population have been to school, and the main economic activities are small-scale agriculture and livestock breeding. In recent years, gold mining in the neighbouring villages of the HDSS has grown in scale. Although the site covers the town of Kaya, health indicators and fertility levels are typical of a rural area of Burkina Faso. Life expectancy was estimated at 54 years in 2013 and the total fertility rate was estimated at 7 children per woman. Households are visited every six months. In case of death, causes of deaths are certified by physicians based on information available in VA questionnaires. During the period considered in this study (2012–2015), a large share of VA questionnaires was not completed and available ones were not yet diagnosed by physicians. However, lay reporting of causes of death was available.

The Mlomp HDSS was set up in 1985 in the Southwest Senegal in the administrative region of Ziguinchor, nearly 500 kilometres from Dakar, the capital city [36]. The site covers 11 villages. The population under surveillance belongs to the Diola ethnic group and is mostly animist or Catholic. Rice cultivation is the main activity in the area but many adults migrate during the dry season, with men leaving to find work in wine palm harvesting and fishing in other regions. Young women are often employed as domestic servants in Dakar or in Banjul (the capital city of Gambia) before they get married. The educational level is relatively high in Mlomp with respect to other rural areas of Senegal. In the 2000s, while only a minority of women aged 15–49 years in rural Senegal as a whole had attended school, around half done so in Mlomp. Health indicators are also encouraging, thanks to a very dynamic private health centre opened in 1961 by French Catholic nurses. However, to see a physician, patients must be referred to the local hospital at Oussouye, 10 kilometres from Mlomp. Advanced medical care including surgery is only available in the larger regional hospital at Ziguinchor, 50 kilometres from Mlomp. The follow-up population was estimated at 9000 inhabitants in 2015 and vital events are updated on an annual basis. In case of death, physicians interpret the completed VA questionnaires to assign a probable cause of death.

### 

#### Variables

Two outcome variables were considered in this analysis. First, place of death was grouped into two main categories: health facility versus non-health facility. We did not make any distinction between the types of health facility; for example, public versus private. The ‘non-health facility’ category mainly included deaths that occurred at home. Deaths that took place elsewhere or for which information was not available represent 5.1% of deaths in Ouagadougou, 2.4% in Kaya and 2.1% in Mlomp.

Second, mobility before death was defined using information on location of death, i.e. if the death has occurred within or outside the HDSS. For the particular case of the Ouagadougou HDSS, to ignore mobility within the city, location of death was defined on the basis of the entire city. Individuals were classified into two categories: those who died in Ouagadougou and those who died elsewhere. Furthermore, for deaths at Mlomp, it was possible to know if the deceased person had left the site to seek health care or not.

Independent variables included sex, age group at death, education, marital status, birthplace and group of causes of death. We categorized the different variables in this way to ensure comparability across the three sites. Age of death was divided into four categories, 15–39, 40–59, 60–79, and 80 years and more. For education, two main categories were considered: individuals with no schooling, and those with at least one year of schooling. We defined three marital-status categories: married, single, divorced/widowed. In some cases, data on education and marital status were missing. These cases were coded as ‘unknown’. Place of birth was taken into account only in the Ouagadougou HDSS, and two categories were defined: native of Ouagadougou and non-native. Finally, causes of death recorded in the sites of Ouagadougou and Mlomp were aggregated into three main categories, excluding deaths from external causes: malaria, HIV/AIDS, respiratory infections and other infections were classified as communicable diseases; diseases such as neoplasms, diabetes, stroke and other chronic diseases were grouped as ‘non-communicable diseases’; and indeterminate causes of deaths and deaths for which there was no VA were classified as ‘ill-defined’.

#### Statistical analyses

In this study, we analyse adult deaths after 15 years of age. The analysis covers the period 2012–2015 in Ouagadougou and Kaya. Since the population of Mlomp is much smaller, the analysis was extended to cover deaths over the period 2000–2015. In Ouagadougou and Kaya, a six-months criterion is used to define residency in the HDSS, i.e. individuals are excluded from the follow-up after six months of absence. This is not the case in Mlomp, due to a high volume of circular migrations. Individuals are excluded from the follow-up only after two successive years of absence. In order to approximate the same residence criteria in Mlomp as in the two other sites, the date of the most recent presence of the residents who died was compared to the date of their death. When the precise date of departure from the village was missing, it was estimated on the basis of information on the person’s presence or absence during the dry and the rainy seasons recorded in the two last follow-up surveys. The deaths of individuals who reported as absent from the HDSS area more than six months are then excluded from the analysis.

As the analysis aims to highlight mobility before death for health reasons, deaths from external causes were excluded (8.4% of deaths in Ouagadougou, 4.5% in Kaya and 9.5% in Mlomp). In the Kaya site, causes of death based on VAs were not available for the period considered, so lay reporting of causes of deaths (disease, accident, suicide, murder, pregnancy-related deaths), by the relatives of the deceased person, was used to exclude deaths from external causes. To sum up, the analysis included 536 eligible deaths in Ouagadougou, 695 in Kaya and 708 in Mlomp. In Mlomp, out of 809 deaths, 101 were discarded because the persons had been away from the site more than six months before their death.

Two sets of analyses were performed for each site. In a first step, in order to investigate the reasons for ultimate mobility, the net effect of the location of death on the place of death (in health structure or not) was assessed using a binary logistic regression. Covariates included sex, age group at death, marital status, education, and group of causes of death. In the second analysis, location of death was the outcome variable to determine factors associated with mobility before death. Its association with independent variables (sex, age group at death, marital status, education, and group of causes of death) was tested again using a binary logistic regression. For the particular case of Ouagadougou, place of birth was also included in the model to examine the effects of migration status (native or not) on location of death. All analyses were performed using STATA software, version 14.

## Results

### Location of death and place of death

The proportion of adults who died outside the HDSS was rather low and similar in the sites of Ouagadougou (5.9%) and Kaya (5.3%). The situation was quite different in Mlomp where 20.6% of deaths occurred outside the HDSS. Regarding the association between place of death and location of death, different pictures emerged for each site (Table 1). In the urban setting of Ouagadougou, 45.7% of deaths occurring in the city took place in health facilities, compared to 19.4% among deaths that occurred outside the city. The reverse situation was observed in the site of Kaya with 34.2% of deaths registered in health facilities among deaths that occurred within the HDSS compared to 70.3% for deaths that occurred outside. In the rural area of Mlomp, the differences in the proportions of health facility deaths by location of death were much larger, with 80.8% of deaths outside Mlomp occurring in health facilities. The figure was only 5.0% for deaths that occurred within the site.

**Table 1.** Proportion of deaths over 15 years of age that occurred in health facilities by location of death in Ouagadougou (2012–2015), Kaya (2012–2015) and Mlomp (2000–2015) HDSSs.

	Ouagadougou HDSS	Kaya HDSS	Mlomp HDSS
**Location of death**			
In the site	45.7%	34.2%	5.0%
Outside the site	19.4%	70.3%	80.8%
**Proportion of deaths occurring in health facilities**	44.1%	36.1%	20.6%

Source: Ouagadougou, Kaya and Mlomp HDSSs

The results of the logistic regression presented in Table 2 confirm the net effect of location of death on place of death in the three sites. This association was adjusted to the independent variables (sex, age group at death, education, marital status, place of birth in Ouagadougou, and group of causes of death in Ouagadougou and Mlomp). In the sites of Mlomp and Kaya, the odds of health facility death were higher for adults who died outside the HDSS than for those who died within the site. These results suggest that mobility before death in the two sites was motivated by health-care-seeking. This assumption is reinforced by the fact that in Mlomp, among those who died outside the site, in 91.1% of cases, their relatives reported that they left the village to seek health care. A contrasting picture emerged in Ouagadougou. The odds of dying in a health facility were lower for adults who died outside the city compared to those who died within the city. So, adults of Ouagadougou who moved before their death were probably not seeking medical care.

**Table 2.** Adjusted odds ratios of the binary logistic regression for the probability that a death occurred in a health facility in Ouagadougou (2012–2015), Kaya (2012–2015) and Mlomp (2000–2015) HDSSs.

	Ouagadougou HDSS		Kaya HDSS		Mlomp HDSS	
**Died^(1)^ outside the site**	OR (95% CI)	p	OR (95% CI)	p	OR (95% CI)	p
No	1		1		1	
Yes	0.2 (0.1–0.5)**	0.002	3.3 (1.5–7.1)**	0.003	77.9 (42.2–143.7)***	<0.001

Source: Ouagadougou, Kaya and Mlomp HDSSs

Statistical significance: ***p < 0.001; **p < 0.05; *p < 0.1

**^(1)^** Adjusted to sex, age group at death, education, marital status, birthplace for Ouagadougou and group of causes of death for Ouagadougou and Mlomp.

### Socio-economic characteristics and location of death

The proportions of deaths outside each site by socio-economic characteristics are shown in Table 3. In the Ouagadougou site, as expected, the proportion of deaths outside the city was twice as high among non-natives (6.9%) as among natives (3.0%) of the city. In the three sites, the proportion of deaths outside the site decreased with age group. Compared to uneducated adults, a higher proportion of those who had at least one year of schooling died outside the sites in Kaya and Mlomp (17.1% vs. 1.9% in Kaya; 37.1% vs. 19.4% in Mlomp). But in Ouagadougou, the proportions of deaths occurring outside the city do not vary much by level of education. Regarding marital status, differences between single and married adults were more noticeable in Kaya and Mlomp, but not in Ouagadougou. Finally, in the sites of Mlomp and Ouagadougou, the proportion of deaths occurring outside each site was higher for deaths due to non-communicable diseases than for deaths from communicable diseases.

**Table 3.** Proportion of deaths over 15 years of age that occurred outside the site by socio-demographic characteristics, and adjusted odds ratio of the binary logistic regression for the probability that a death occurred outside the site, in Ouagadougou (2012–2015), Kaya (2012–2015) and Mlomp (2000–2015) HDSSs.

	Ouagadougou HDSS			Kaya HDSS^(1)^			Mlomp HDSS^(2)^		
Variables	%	OR (95% CI)	p	%	OR (95% CI)	p	%	OR (95% CI)	p
**Birthplace**									
Natives	3.0	1							
Non-natives	6.9	2.9 (1.0–8.8)*	0.056						
**Sex**									
Male	4.5	1		6.2	1		21.2	1	
Female	7.6	2.0 (0.8–4.7)	0.131	4.4	1.0 (0.4–2.1)	0.956	15.5	0.9 (0.5–1.3)	0.504
**Age group at death**									
15–39	8.9	1		17.2	1		45.5	1	
40–59	7.2	0.7 (0.2–1.9)	0.450	5.3	0.4 (0.2–1.1)	0.083	33.3	0.3 (0.1–0.7)**	0.007
60–79	3.6	0.2 (0.1–0.8)**	0.019	2.0	0.2 (0.0–0.5)**	0.002	18.4	0.1 (0.0–0.3)***	<0.001
80+	4.3	0.2 (0.0–1.1)*	0.069	1.9	0.2 (0.0–0.7)**	0.015	6.3	0.0 (0.0–0.1)***	<0.001
**Education**									
None	5.8	1		1.9	1		15.5	1	
Primary +	5.8	0.7 (0.3–1.7)	0.405	17.1	5.3 (1.8–16.0)**	0.003	37.1	1.2 (0.6–2.3)	0.633
Unknown	12.5	1.7 (0.2–17.2)	0.641	10.3	–	0.990	19.4	1.0 (0.6–1.5)	0.843
**Marital status**									
Married	5.5	1		2.5	1		24.2	1	
Single	7.6	1.2 (0.3–4.6)	0.755	17.9	1.6 (0.5–5.5)	0.453	28.1	0.3 (0.1–0.8)*	0.010
Divorced/Widowed	6.2	1.1 (0.4–3.1)	0.888	2.2	2.3 (0.6–9.7)	0.245	11.6	0.6 (0.4–1.0)*	0.053
Unknown				11.1	–		16.7	0.5 (0.1–3.5)	0.504
**Group of causes of death**									
Communicable diseases	3.2	1					16.7	1	
Non-communicable diseases	7.2	3.4 (1.3–9.0)**	0.014				20.2	1.2 (0.7–2.0)	0.451
Ill-defined	8.7	3.6 (1.0–14.4)*	0.064				16.9	1.1 (0.6–2.0)	0.839
Total	5.9			5.3			20.6		
**Number of deaths**	**536**			**695**			**708**		

Source: Ouagadougou, Kaya and Mlomp HDSSs

Statistical significance: ***p < 0.01; **p < 0.05; *p < 0.1

^(1)^ In Kaya, people with unknown education and marital status are similar.

^(2)^ In the site of Mlomp, including a dummy variable in the model to control the effect of the period 2012–2015 does not change the results. The period does not have a significant effect at the 10% level.

The adjusted odds ratio of the effects of the independent variables on the location of death are presented in Table 3. For the particular case of Mlomp, results taking into account reasons for mobility before death are presented in the appendix (Table A1). Whether ‘death outside the village due to health care seeking’, or simply ‘location of death’, is used as the outcome variable, the results were similar. In the urban HDSS of Ouagadougou, the odds of dying outside for non-natives of the city were three times those of natives. Adult deaths due to non-communicable diseases were more likely to take place outside the city than deaths due to communicable diseases. The same goes for ill-defined deaths. However, in the rural site of Mlomp, there was no significant association between broad group of causes of death and location of death.

The age group had the strongest association with the location of death. Whatever the site, elderly people were much less mobile before their death than young adults (aged below 40 years). In Kaya and Mlomp, there was a marked difference between people who died before age 40 and those who died at older ages. In the case of Ouagadougou, a significant association was found only between those aged 60 and more, and young adults.

Education does not affect location of death in either Ouagadougou or Mlomp. A strong effect was observed in Kaya, however. The odds of dying outside the site for adults who had at least one year of schooling was five times that of those with no schooling. The effect of marital status on location of death was only significant in the site of Mlomp.

## Discussion

In this paper, we document the magnitude of short-term mobility before death in rural, semi-rural and urban areas of Burkina Faso and Senegal, along with the reasons for this mobility and the associated factors. The analysis was based on recent data on deceased adults (aged 15 years and above) collected in three HDSS sites (Ouagadougou, Kaya, Mlomp). To the best of our knowledge, this is the first time that short-term mobility before death was investigated in West Africa.

First of all, the results indicate that in rural and semi-rural areas, location of death is a good indicator for identifying health-care-seeking mobility at the end of life. Adults who moved outside their usual place of residence shortly before their death were clearly looking for medical care. Nonetheless, the magnitude of mobility depends on the supply of health facilities in each site. In Kaya, the supply of health facilities is acceptable; it includes a regional hospital, which is a referral centre for surrounding villages. This explains why the proportion of adults dying outside the area is rather low (5.3%). It is likely that adults who moved before their death were too seriously ill to be treated in Kaya. They were probably looking for specialized care in a larger city such as Ouagadougou, which is not far away. In the site of Mlomp, on the other hand, the supply of health services consists solely of one primary health-care facility with a nurse and a maternity clinic. Since the area mostly provides mother and child health care, it is not surprising that seriously ill adults seek health care outside the village. The nearest hospitals in the region are in the small city of Oussouye, located 10 km from the HDSS area (presence of a physician), and in the larger city of Ziguinchor located 50 km from the HDSS area (more specialized care, including surgical facilities). But some people may travel to distant cities, especially Dakar, where they have relatives, to benefit from the family environment and seek medical care.

Our findings also show that adults in urban areas also move before their death despite the relative availability of health care. For example, the level of mobility found in the sites of Kaya (5.3%) and Ouagadougou (5.9%) are similar. But the reasons behind these mobility flows may be different. Our results showed that deaths outside Ouagadougou were more likely to occur at home than deaths that took place in the city. Furthermore, the supply of health facilities is better in the city than in other locations in Burkina Faso [34]. It is likely that urban dwellers who moved before death did not have easy access to or desperately used the existing health care. Then, they preferred to find relief and comfort among their relatives in their home community. This was particularly the case for the non-natives of the city. The results confirmed the hypothesis that non-natives of the city were more likely to die outside the city than natives. It is quite probable that they went back to rural areas. In the Ouagadougou HDSS, around 70% of non-native adults over 15 years of age were born in rural areas [33].

In rural and semi-rural areas, age was strongly associated with mobility before death, with younger adults being more mobile than elderly people. This is consistent with the fact that functional limitations tend to increase with age and hinder elderly people’s access to medical care [37,38]. Their mobility may also be compromised by the lack of support from close relatives, especially the helping hand of young adults who are generally involved in rural–urban migration. In addition to disability issues, cultural barriers also play a key role in explaining the decrease in health-care-seeking with age. Previous studies in Mlomp have found that older adults are generally more reluctant to use health facilities and prefer to rely on traditional medicine [18]. Furthermore, in the context of poverty, ageing per se is rapidly conceptualized as a cause of death in itself. The community and family members are convinced that any treatment in favour of elderly people will be ineffective. Sometimes, they prefer to invest more money in their funeral to ensure that their soul rests in peace [39]. Finally, older adults see themselves as unproductive and as a burden for their close relatives. For example, it has been reported in Senegal and Burkina Faso that after a certain age, some prefer to free themselves through death [18,39].

Regarding the causes of death, deaths due to non-communicable diseases were more likely to occur outside Ouagadougou than deaths due to communicable diseases. But, in the rural setting of Mlomp, we did not find a significant association between causes of death and mobility before death. Findings in Ouagadougou suggest that even in some cities, access to health care for non-communicable diseases remains problematic. First, in the context of a poor country such as Burkina Faso, it is likely that the supply of specialized services for patients with these diseases is limited. Globally, health systems in West Africa continue to focus on infectious diseases despite global warnings on the rise of non-communicable diseases [40]. Second, because of lack of a palliative care, patients are obliged to develop coping strategies at the end of their life. It is probable that those suffering from chronic diseases have sufficient time to travel. Finally, cultural barriers or poverty can compromise patients’ demand for health services. In particular, treatment of non-communicable diseases is generally costly in contexts where individuals have no health insurance [22].

In Mlomp, several mechanisms may explain the absence of differences in mobility before death between the groups causes of death. The concept of non-communicable diseases is not well understood in rural settings of Africa [41]. Rural residents are used to dealing with infectious diseases and may continue to resort to the same therapeutic itinerary, even in the cases of non-communicable diseases. A striking example is that of patients with hypertension and diabetes. It is reported that few are aware of their disease and even fewer seek care because of the lack of symptoms and the high costs of treatment [42,43]. In rural settings, health-care-seeking may perhaps also be seriously compromised by lack of income. Patients need to have sufficient resources to undertake a difficult journey to seek care outside their place of residence.

In addition to age and group of causes of death, other variables such as education and marital status play a role in determining mobility before death in rural and semi-rural areas. While in Mlomp single adults are less mobile before their death than married ones, no effect was observed in Kaya. Consistent with some previous findings in SSA [11,12], married adults can rely on the support of their partner to access medical care. But the effect of marital status may be blurred by the lack of information on standards of living. Married adults are likely to be wealthier than single adults. The confusion between the effects of marital status and standard of living may be higher in Kaya than in Mlomp. In this site, one can expect mobility before death to be more selective. As there is already a regional hospital in the area, it may be the most educated and wealthy residents who tend to travel to larger cities such as Ouagadougou to access the best health-care services. This is confirmed by the fact that adults who went to school tend to be more mobile before their death in Kaya, whereas level of education had no impact in Mlomp.

It is important to point out various limitations of this study. First, in the three sites, deaths from external causes were excluded using different methods. However, in the West African context, sociability is so strong that these deaths generally do not go unnoticed in the community. It is thus likely that the different methods used to determine causes of deaths yield consistent results regarding deaths from external causes. Second, comparisons of causes of death in Mlomp and Ouagadougou are limited in this study because these causes are diagnosed using different methods (physician certification in Mlomp and interVA-4 software in Ouagadougou). We cannot guarantee that interpretation of VAs and mobility before death are independent, particularly when VAs are reviewed by physicians. In Mlomp, the illness history section of the VAs may include some information on the patient’s mobility before death that might influence the physician’s interpretation. But the illness history is not taken into account in the inter-VA-4 software [31]. Previous research has shown ‘a moderate agreement’ between causes of death derived from the inter-VA software and diagnosed by physicians [44,45]. Furthermore, in this study, inconsistency between the two methods of cause of death interpretation may be limited by the use of broad groups of causes of death. Third, in rural and semi-rural areas, it was not possible to distinguish between patient-initiated mobility and emergency referral to another hospital without passing through a relative’s home. Such a distinction would refine the analysis. However, the reasons for mobility reported in Mlomp for deaths outside the site suggest that cases of emergency referral were limited. Four, representativeness of HDSSs cannot be guaranteed in this study. The different sites included may not represent the full diversity of urban and rural settings of West Africa. Finally, the effects of some variables on mobility before death may be biased by the lack of some information. Adults’ standards of living, and, for rural residents particularly, the existence of strong social ties in cities, can influence mobility before death.

## Conclusion

Despite these limitations, this study paves the way for future research on mobility before death in rural and urban areas of West Africa. Considering the evolutionary stages in societies related to place of death conceptualized by Gu et al. [1], our findings show that many rural residents and urban residents of rural origin cannot obtain both medical and supportive care in the same place. Depending on their capacities (economic, networking, physical), they will develop coping strategies. Some rural residents leave home to seek health care, and some urban residents, particularly those with rural origins, return home to die. The two types of mobility do not necessarily occur at the same stage of the end of life: one is motivated by the hope of a cure and the other by the end of this hope. In this context, some individuals may be involved in circular mobility at the end of their life. Of public health importance, the movements of terminally ill urban and rural residents can affect the estimation of urban/rural mortality differentials. The death of an adult who has moved away from home may be wrongly classified as an urban or rural death. This is likely to occur in large data collection operations such as censuses which focus on the number of deaths occurring in each household during the last 12 months.

## Paper context

In sub-Saharan Africa, the literature on end of life focuses on place of death. Little is known about population mobility at the end of life. This study documents short-term mobility before death among adults over 15 years of age in Burkina Faso and Senegal. Some rural residents move to access health care while some urban dwellers move to benefit from supportive care. These movements preceding death can affect the estimation of urban/rural mortality differentials.

## Appendix

**Table A1.** Adjusted odds ratio of the binary logistic regression on the probability that a death occurred outside the site of Mlomp due to health-care-seeking (2000–2015).

Variables	OR (95% CI)	P values
**Sex**		
Male	1	
Female	0.9 (0.6–1.4)	0.603
**Age group at death**		
15–39	1	
40–59	0.3 (0.1–0.8)**	0.015
60–79	0.2 (0.1–0.4)***	<0.001
80+	0.1 (0.0–0.1)***	<0.001
**Education**		
None	1	
Primary +	1.1 (0.6–2.3)	0.694
Unknown	1.0 (0.6–1.5)	0.879
**Marital status**		
Married	1	
Single	0.3 (0.1–0.8)**	0.013
Divorced/Widowed	0.7 (0.4–1.1)	0.105
Unknown	0.2 (0.0–2.4)	0.221
**Group of causes of death**		
Communicable diseases	1	
Non-communicable diseases	1.2 (0.7–2.0)	0.592
Ill-defined	1.0 (0.5–2.0)	0.908
**Number of observations**	708	

Source: Mlomp HDSS

Statistical significance: ***p < 0.01; **p < 0.05; *p < 0.1
